# Electrocardiographic Findings and Clinical Outcome in Patients with COVID-19 or Other Acute Infectious Respiratory Diseases

**DOI:** 10.3390/jcm9113647

**Published:** 2020-11-12

**Authors:** Antonio De Vita, Salvatore Emanuele Ravenna, Marcello Covino, Oreste Lanza, Francesco Franceschi, Filippo Crea, Gaetano Antonio Lanza

**Affiliations:** 1 Department of Cardiovascular and Thoracic Sciences, Fondazione Policlinico Universitario A. Gemelli IRCCS, Università Cattolica del Sacro Cuore, 00168 Rome, Italy; antonio.devita90@gmail.com (A.D.V.); e91.rav@gmail.com (S.E.R.); filippo.crea@unicatt.it (F.C.); 2 Department of Emergency Medicine, Fondazione Policlinico Universitario A. Gemelli IRCCS, Università Cattolica del Sacro Cuore, 00168 Rome, Italy; macovino@gmail.com (M.C.); francesco.franceschi@unicatt.it (F.F.); 3 Department of Clinical and Molecular Medicine and Psychology, Università La Sapienza, 00189 Rome, Italy; orelanza@gmail.com

**Keywords:** acute infectious respiratory disease, SARS-CoV-2 infection, COVID-19, electrocardiogram, clinical outcome

## Abstract

Background. Cardiac involvement in coronavirus SARS-CoV-2 infection (COVID-19) has been reported in a sizeable proportion of patients and associated with a negative outcome; furthermore, a pre-existing heart disease is associated with increased mortality in these patients. In this prospective single-center case-control study we investigated whether COVID-19 patients present different rates and clinical implications of an abnormal electrocardiogram (ECG) compared to patients with an acute infectious respiratory disease (AIRD) caused by other pathogens. Methods. We studied 556 consecutive patients admitted to the emergency department of our hospital with symptoms of AIRD; 324 were diagnosed to have COVID-19 and 232 other causes of AIRD (no-COVID-19 group). Standard 12-lead ECG performed on admission was assessed for various kinds of abnormalities, including ST segment/T wave changes, atrial fibrillation, ventricular arrhythmias, and intraventricular conduction disorders. Results. ECG abnormalities were found in 120 (37.0%) and 101 (43.5%) COVID-19 and no-COVID-19 groups, respectively (*p* = 0.13). No differences in ECG abnormalities were found between the 2 groups after adjustment for clinical and laboratory variables. During a follow-up of 45 ± 16 days, 51 deaths (15.7%) occurred in the COVID-19 and 30 (12.9%) in the no-COVID-19 groups (*p* = 0.39). ST segment depression ≥ 0.5 mm (*p* = 0.016), QRS duration (*p* = 0.016) and presence of any ECG abnormality (*p* = 0.027) were independently associated with mortality at multivariable Cox regression analysis. Conclusion. Among patients hospitalized because of AIRD, we found no significant differences in abnormal ECG findings between COVID-19 vs. no-COVID-19 patients. The ECG on admission was helpful to identify patients with increased risk of death in both groups of patients.

## 1. Introduction

SARS-CoV-2 is a novel coronavirus that is causing a pandemic outbreak of respiratory disease from the end of 2019 (COVID-19) [1,2]. Bilateral interstitial pneumonia is the most typical manifestation of COVID-19 and can be complicated by acute respiratory distress syndrome, multiorgan failure and death in up to 30% in high-risk patients [3,4].

The risk of severe complications and death has been found to be increased in male elderly subjects, in particular in presence of underlying co-morbidity, including hypertension, diabetes and cardiovascular disease [5,6,7]. Importantly, a few studies have shown that COVID-19 is associated with myocardial injury in about 20–30% of hospitalized patients, which portends an increased risk of negative outcome [8,9,10,11,12,13,14]. However, it is not known whether the prevalence of cardiac involvement is increased in COVID-19 as compared to other acute infectious respiratory diseases (AIRDs), which have also been reported to be associated with myocardial injury in a sizeable proportion of patients [15,16].

The electrocardiogram (ECG) is the most simple and available method to screen for the possible presence of cardiac abnormalities. Of note, in a recent study we found that the ECG performed on admission predicted 30 day mortality in COVID-19 patients [17]. To the best of our knowledge, however, no previous study assessed the prognostic value of ECG in AIRDs unrelated to SARS-CoV-2 infection.

In this study we aimed to: (1) Evaluate whether patients with a diagnosis of COVID-19 present a different rate of ECG abnormalities, suggesting cardiac involvement, compared to patients with other causes of AIRD; (2) assess the prognostic implications of ECG in patients hospitalized because of an AIRD, as well as whether there are differences in the prognostic implications of ECG in patients with or without COVID-19.

## 2. Materials and Methods

We studied consecutive patients with age >20 years admitted to the Emergency Department of our University Hospital (Fondazione Policlinico Universitario A. Gemelli IRCCS, Rome, Italy) because of symptom of AIRD and suspicion of COVID-19, between the 1 March and 15 April 2020. All patients had been referred because of fever unresponsive to anti-pyretic drugs and symptoms possibly consistent with COVID-19 (e.g., cough, dyspnea, tachypnea, sputum production).

COVID-19 testing was based on the protocol released by the World Health Organization (WHO) [18]. Nasopharyngeal swab specimens were collected in all patients and SARS-CoV-2 RNA was detected by reverse-transcription polymerase chain reaction (RT-PCR). Chest X-ray and, when indicated, thoracic computerized tomography (CT) scan were performed to confirm the diagnosis.

Patients were divided in 2 groups based on the diagnosis of the AIRD: (1) COVID-19 group and (2) no-COVID-19 group, including all patients with any other infectious cause of the AIRD. Patients in the COVID-19 group were those included in our recent study on the prognostic value of the ECG in these patients [17], for whom an extended follow-up is reported in the present study.

The demographic characteristics (age and sex), clinical data and laboratory findings on admission were acquired from our Institutional database. When available, cardiac troponin I (cTnI) and N-terminal pro-B-type natriuretic peptide (NT-proBNP) serum levels were also acquired. The upper normal level for cTnI for our laboratory was 39 ng/L, whereas the upper normal level for NT-proBNP was 450 pg/mL.

A history of known heart disease included any evidence of coronary artery disease (previous myocardial infarction, coronary revascularization and/or documented obstructive coronary stenosis at angiography), heart failure, moderate to severe valvular heart disease or cardiomyopathy, as documented in patients’ clinical reports. The presence of pre-existing comorbidities, including chronic obstructive pulmonary disease (Gold stage 3-4), severe renal failure (stage 4-5), stroke in the previous 12 months, chronic inflammatory disease, and neoplasia, was also recorded. The study was approved by the Ethics Committee of our Institution. All patients admitted to the Emergency Department signed a comprehensive ethical agreement for the collection of blood samples and clinical data, for bio-bank and research purposes.

### 2.1. ECG Analysis and Definitions

Twelve-lead standard ECGs were recorded on admission with a Mortara ELI 350 ECG machine (Mortara Instrument Europe, Bologna, Italy). The ECGs were retrieved by our dedicated Institutional ECG storage server (Mortara X-scribe; Mortara S.p.A. Bologna, Italy) and independently analyzed by two trained fellows in cardiology (A.D.V., S.E.R.). Discordances were solved by consensus, with the supervision of the senior expert in electrocardiography (G.A.L.). The ECGs were analyzed before proceeding with assessment of clinical outcome, which was therefore unknown to the ECG readers.

As previously described [17], the ECGs were analyzed for the following parameters: rhythm, presence of atrio-ventricular blocks, complete right (RBBB) or left (LBBB) bundle branch block, ST-segment and/or T wave abnormalities suggesting myocardial ischemia or injury, abnormal Q/QS waves typical of a previous myocardial infarction. The presence of premature supraventricular (PSVCs) and ventricular (PVCs) complexes was also recorded.

The PR and QT intervals and QRS duration were derived from the automatic measurements of the Mortara X-Scribe software, after checking for their reliability. Patients with LBBB were excluded from ST-segment and T wave analyses, whereas in patients with RBBB, only leads V1-V4 were excluded from ST-segment and T wave analysis.

ST segment depression (STD) was diagnosed when a horizontal or downsloping displacement of the ST segment below the isoelectric line ≥ 0.5 mm, persisting at 0.08 s from the J point, was detectable in at least two contiguous leads. A separate analysis was also conducted considering as abnormal an STD ≥ 1 mm. ST-segment elevation (STE) was diagnosed when the J point was elevated by ≥1 mm and morphology was judged to be compatible with an ischemic or pericarditis origin. An abnormal T wave was diagnosed in case of T wave inversion ≥ 1 mm in at least two contiguous leads (except V1 and aVR). The combined presence of STD ≥ 0.5 mm and/or T wave inversion (STD_0.5_/T changes) and STD ≥ 1 mm and/or T wave inversion (STD_1.0_/T changes) was also considered.

The QT interval was corrected for heart rate (cQT) using Bazett’s formula (cQT = QT(ms)/√RR(s)). In patients with QRS duration ≥ 120 ms, however, the cQT was calculated using the formula validated by Rautaharju et al.:

cQT_RR−QRS_ = QT − 155 × (60/HR − 1) − 0.93 × (QRS − 139) + k (1)

where k = −22 ms for men and −34 ms for women, respectively [19].

The presence of any ECG abnormality was defined as the presence of one or more of the following abnormalities: Non-sinus rhythm; ≥II degree atrio-ventricular block; QRS duration ≥ 110 ms, STD or STE, negative T wave, cQT ≥ 460 in women or ≥450 in men [20]; pathologic Q/QS wave; presence of PSVCs or PVCs.

### 2.2. Clinical Outcome

The vital status of patients was determined on 10th of May 2020 by consulting the clinical database of our hospital and, in case of patient discharge, by telephone call.

### 2.3. Statistical Analysis

Data are reported as mean and standard deviation for continuous variables and number and proportions for discrete variables. Continuous variables were compared by analysis of variance, whereas proportions were compared by Fisher exact test or chi-square test, as indicated. Differences between the 2 groups in ECG variables were adjusted for clinical and laboratory variables that showed a significant or borderline (*p* ≤ 0.1) statistical difference between the 2 groups, using multivariable logistic regression for discrete variables and a generalized linear model for continuous variables.

Univariable and multivariable survival analyses for global mortality were performed by Cox regression. Only variables with a *p*-value ≤ 0.1 at univariable analysis were included in the multivariable models. Multivariable analysis was first performed on clinical and laboratory variables. ECG variables were then individually added to the identified independent clinical/laboratory variables to assess whether they maintained a significant association with mortality. Survival curves were derived with the Kaplan–Meier method and compared by log-rank test.

Since cTnI and NT-proBNP were available for 16.4% and 20.7% of patients only, they were not included in the general survival analyses. However, the association of increased levels of cTNI and NT-proBNP with death was assessed in the subgroups with available data; furthermore, we tested whether predictive ECG variables maintained an independent association with death after adjustment for elevated cTnI and NT-proBNP levels using multivariable Cox regression.

A *p* < 0.05 was always required for statistical significance. Data were analyzed by the SPSS 21.0 statistical software (SPSS Italia, Inc., Florence, Italy).

## 3. Results

### 3.1. General Findings

**Figure 1:**
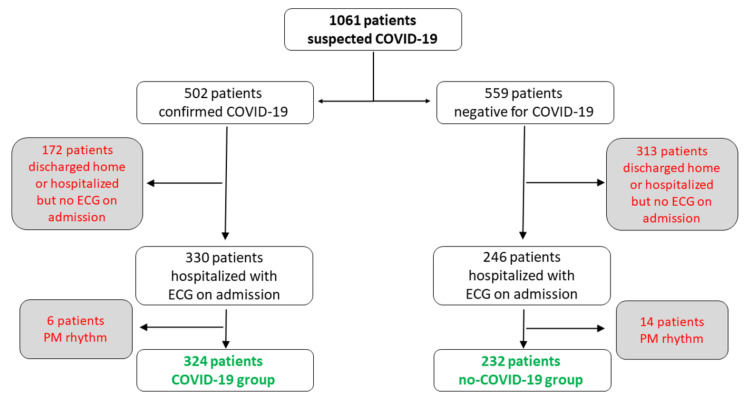
Flow-chart of patients’ enrolment. COVID-19 = coronavirus disease 2019; ECG = electrocardiogram; PM = pacemaker.

A flow-chart of patients’ enrolment is shown in Figure 1. Overall, 1061 patients, referred to our Emergency Department because of an AIRD, were considered for the study. The diagnostic workout confirmed the diagnosis of COVID-19 in 502 patients (47.3%), whereas the other 559 patients (52.7%) were diagnosed to have other causes of AIRD. Standard ECG on admission was available for 576 patients, 330 (57.3%) in the COVID-19 group and 246 (42.7%) in the no-COVID-19 group. Six patients (1.8%) of COVID-19 group and 14 patients (5.7%) of no-COVID-19 group were excluded because of a pacemaker rhythm, which precluded a reliable assessment of spontaneous cardiac electrical activity. Thus, the study population eventually included 324 COVID-19 patients and 232 no-COVID-19 patients. The main clinical characteristics of the 2 groups of patients are summarized in Table 1. COVID-19 patients were younger (*p* = 0.033) and included a higher proportion of male (*p* = 0.008) compared to no-COVID-19 patients. Peripheral capillary oxygen saturation (SpO_2_) was significantly lower in COVID-19 patients (*p* < 0.001), but a history of heart disease (*p* = 0.021) and co-morbidity (*p* < 0.001) were significantly more frequent in the no-COVID-19 group. Among patients with available data, NT-pro-BNP levels were higher in no-COVID-19 patients (*p* = 0.008), whereas no difference was observed in cTnI levels (*p* = 0.22).

### 3.2. ECG Findings

The main ECG findings of the 2 groups of patients are summarized in Table 2. As shown, any ECG abnormality was found in 120 COVID-19 patients (37%) and in 101 (43.5%) patients with other AIRD diagnoses (*p* = 0.13). Most individual ECG abnormalities were similar in the 2 groups, including ST-segment and T wave changes, a QRS ≥ 110 ms, a prolonged cQT interval and the rate of ventricular arrhythmias. In particular, while no patient showed pathologic STE, the rate of STD did not significantly differ between the 2 groups, either when a cut-off level ≥ 0.5 mm (*p* = 0.68) or ≥ 1 mm (*p* = 0.13) was considered.

COVID-19 patients, compared to no-COVID-19 patients showed a lower rate of atrial fibrillation (*p* = 0.021), LBBB (*p* = 0.011) and PSVCs (*p* = 0.029), as well as a lower cQT interval (*p* = 0.03). These differences, however, lost statistical significance after adjustment for the main clinical/laboratory variables (Table 2).

### 3.3. ECG Findings and Clinical Outcome

**Figure 2:**
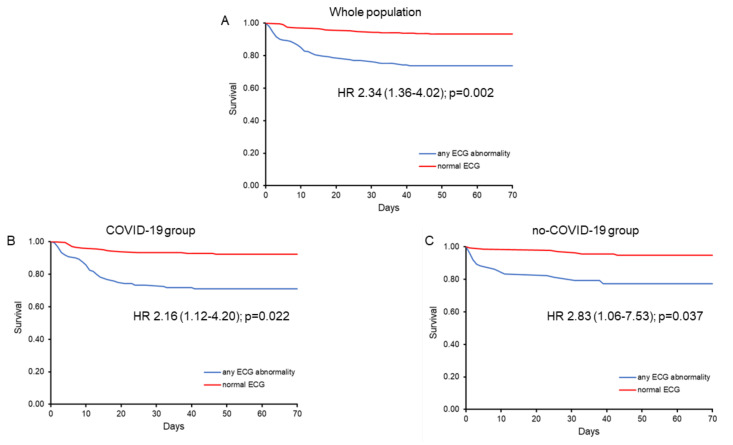
Kaplan–Meier survival curves of patients with and without the presence of any electrocardiogram abnormality in the whole population of patients hospitalized because of an acute infectious respiratory disease (**A**) and in the COVID-19 (**B**) and no-COVID-19 (**C**) groups. ECG = electrocardiogram; HR = hazard ratio (with 95% confidence interval).

During a follow-up of 45 ± 16 days (range 1–70), 81 deaths (14.6%) occurred in the whole population of AIRD patients, 51 (15.7%) in the COVID-19 group and 30 (12.9%) in the no-COVID-19 group (HR 1.22; 95% CI 0.77–1.91; *p* = 0.39). Many clinical and ECG variables were significantly associated with death at univariate analysis (Table 3). Clinical and laboratory variables independently associated with death at multivariable analysis, however, included age, co-morbidity, SpO_2_, and creatinine and C-reactive protein (CRP) serum levels. The ECG variables that remained associated with death when individually added to these variables included QRS duration (*p* = 0.016), STD ≥ 0.5 mm (*p* = 0.016) and the presence of any abnormality on the ECG (*p* = 0.002; Figure 2) (Table 4).

In a subgroup analysis of 91 patients with measurement of cTnI serum levels, including 13 deaths (14.3%), elevated cTnI was associated with increased mortality (*p* = 0.006; Table 3). STD ≥ 0.5 mm (HR 4.27; 95% CI 1.11–16.4; *p* = 0.034) and STD_0.5_/T changes (HR 8.02; 95% CI 2.46–26.2; *p* = 0.001) remained independently associated with mortality after adjustment for elevated cTnI levels.

Furthermore, among 115 patients with NT-proBNP serum levels, including 34 deaths (29.6%), elevated NT-proBNP was associated with increased mortality (*p* = 0.001; Table 3). STD ≥ 0.5 mm (HR 3.28; 95% CI 1.48–7.26; *p* = 0.003), STD_0.5_/T changes (HR 3.66; 95% CI 1.73–7.73; *p* = 0.001), QRS duration (HR 1.02; 95% CI 1.01–1.03; *p* = 0.005) and QRS duration ≥ 110 ms (HR 2.26; 95% CI 1.12–4.52; *p* = 0.022) remained independently associated with mortality after adjustment for elevated NT-proBNP serum levels.

A separate multivariable Cox regression analysis in COVID-19 and no-COVID-19 groups revealed some differences between the 2 groups in the ECG variables independently associated with death. LBBB (*p* = 0.023), QRS duration (*p* = 0.003), QRS duration ≥ 110 ms (*p* = 0.044) and STD_0.5_/T wave abnormality (*p* = 0.034) emerged as independent predictors of death in the COVID-19 group, whereas only the presence of STD, both ≥ 0.5 mm (*p* = 0.004) or ≥ 1.0 mm (*p* = 0.010), emerged as an independent predictor of negative outcome in the no-COVID-19 group (Table 5). The presence of any abnormality on the ECG, however, remained associated with death at multivariable analysis both in the COVID-19 (*p* = 0.022) and the no-COVID-19 (*p* = 0.037) group (Table 5 and Figure 2).

## 4. Discussion

The main data emerging from our study can be summarized as follows: (1) Patients with AIRD symptoms hospitalized because of COVID-19 did not show significantly different rates of ECG abnormalities on admission as compared to patients with AIRD caused by other pathogens; (2) the ECG on admission was helpful to identify patients at increased risk of a negative clinical outcome in the whole population of patients hospitalized because of an AIRD, as well as in the COVID-19 and no-COVID-19 subgroups, separately; and (3) there were some differences in the individual ECG variables associated with a negative outcome in patients with COVID-19 as compared with those with other forms of AIRD, suggesting some possible different type of cardiac involvement of the 2 groups.

SARS-CoV-2 infectious disease (COVID-19) is causing a dramatic pandemia, with clusters of elevated mortality related to diffuse acute interstitial pneumonia and severe respiratory distress syndrome [2,3,4,5,6,21]. However, some studies have suggested that 20 to 30% of COVID-19 patients have evidence of cardiac injury, as assessed by increased cTnI serum levels and arrhythmias, and that this is associated with a worse clinical outcome [8,9,10,11,22,23,24]. Of note, a localization of the virus RNA in myocardial cells has been found in a sizeable proportion of deceased COVID-19 patients [25]. However, whether COVID-19 is associated with an increased rate of cardiac involvement, as compared to other kinds of AIRDs, is unknown.

In this study we found that the prevalence of ECG abnormalities was similar in COVID-19 patients and those with a similar AIRD clinical presentation, requiring hospitalization, but negative COVID-19 tests. This concerned both ECG abnormalities compatible with either acute coronary syndromes, such as regional ST-segment elevation or depression [26], or myocardial inflammatory involvement, such as diffuse ST-T wave changes, as well as ventricular arrhythmias and conduction disorders [27]. Thus, our data suggest that, at least in the early phase of the disease, COVID-19 is unlikely to be associated with an increased rate of myocardial suffering or injury, as compared to AIRDs caused by other agents.

The lack of significant differences in an acute cardiac involvement between the 2 groups of AIRD patients was also supported, in our study, by the lack of differences in cardiac troponin I levels, although the latter were obtained only in a selected subgroup of patients. Accordingly, this finding suggests that myocardial injury in these patients depends more on the severity than on the cause of the infectious disease [28].

Of note, among patients who underwent measurement of NT-proBNP levels, elevated concentrations were also found in a similar proportion of COVID-19 and no-COVID-19 patients, although median levels were higher in the no-COVID-19 population, likely because of a higher prevalence of pre-existing cardiac diseases.

Importantly, the presence of ECG abnormalities predicted a worse clinical outcome in our patients, both in the whole population, as well as, separately, in the COVID-19 and no-COVID-19 cohorts. Of note, ST/T wave abnormalities remained predictors of death independently of increased cTnI and NT-BNP levels when survival analyses were performed in the subgroups of patients with available measurements of these 2 markers of cardiac injury/suffering.

We have already reported that ECG predicted short-term clinical outcome in the same population of COVID-19 patients included in this study [17], and this finding is confirmed by the present study in which clinical follow-up was prolonged, on average, from 31 to 45 days. Notably, by extending the follow-up duration, STD ≥ 0.5 mm and/or T-wave inversion (≥1 mm), that previously failed to achieve a significant association with outcome at multivariable analysis [17], were now found to be independent predictors of death in the COVID-19 cohort. Of note, to the best of our knowledge, our study is the first one that reports on the prognostic value of admission ECG in patients with AIRD caused by other microbial agents.

Interestingly, while the presence of any ECG abnormality was an independent predictor of death both in COVID-19 and no-COVID-19 patients, we found some differences between the 2 groups in the individual ECG variables associated with mortality. Intraventricular conduction disorders, as indicated by a prolonged QRS duration or a typical LBBB, were the strongest ECG variable associated with mortality in the COVID-19 patients, in agreement with our previous report [17]. In contrast, intraventricular conduction delay did not emerge as a predictive variable in the no-COVID group, in which the presence of STD, suggesting an acute ischemic injury, was, instead, the only ECG variable independently associated with death. However, whether this difference between the 2 groups indicates true different prognostically relevant involvement of the intraventricular conduction system in COVID-19 patients, as compared to no-COVID patients, or it is merely related to chance remains to be clarified.

## 5. Limitations of the Study

Some limitations of our study should be acknowledged. First, we did not have previous ECGs of patients and, therefore, we could not distinguish between new-onset vs. chronic ECG abnormalities; however, there was no individual ECG finding that showed an increased prevalence in COVID-19 patients, including those more likely related to an acute cardiac involvement (e.g., ST-segment/T wave changes, ventricular arrhythmias, conduction disorders). Second, our data only refer to patients admitted to hospital who underwent an ECG recording on admission. Thus, they may not reflect findings in the whole population of both groups of patients, as it is likely that patients who underwent ECG recording had more severe forms of the disease. Third, we only assessed ECGs on admission; thus, we cannot exclude that a later cardiac involvement with subsequent ECG changes might have been resulted in some differences between the 2 groups. Fourth, we included in the no-COVID-19 group any AIRD not attributable to SARS-CoV-2 infection; therefore, we cannot establish whether differences exist in the rate of ECG abnormalities among this heterogeneous group of patients according to the microbial agent responsible for the AIRD. Finally, we did not have detailed data about pharmacological therapy of our patients and, therefore, we could not establish how treatment influenced ECG findings and clinical outcome [29,30].

## 6. Conclusions

In conclusion, our data show that among patients presenting with fever and symptoms suggesting an AIRD and requiring hospitalization, standard ECG does not seem to show significant differences between those with or without COVID-19. Importantly, The ECG on admission seems helpful to identify patients at increased risk of death among the whole population of AIRD patients as well as, separately, in the groups of COVID-19 and no-COVID-19 patients.

## Figures and Tables

**Table 1:** Main clinical characteristics of the 2 groups of patients.

	COVID-19 Group (*n* = 324)	No-COVID-19 Group (*n* = 232)	*p*-Value
Age (years)	65.9 ± 15	68.9 ± 18	0.033
Male sex (%)	214 (66.0)	127 (54.7)	0.008
Known heart disease (%)	67 (20.7)	68 (29.3)	0.021
Co-morbidity (%)	102 (31.5)	157 (67.7)	<0.001
Hypertension (%)	169 (52.2)	122 (52.6)	0.93
Diabetes mellitus (%)	37 (11.4)	33 (14.2)	0.37
Dyslipidemia (%)	67 (20.7)	45 (19.4)	0.75
Smoking (%)	86 (26.5)	60 (25.9)	0.92
Hemoglobin (g/dL)	13.8 ± 1.7	12.2 ± 2.4	<0.001
Creatinine (mg/dL)	1.21 ± 1.3	1.28 ± 1.3	0.51
C-reactive protein (mg/L) *	77.3 (26.6–152.3)	78.7 (17.2–160.0)	0.42
NT-proBNP (pg/mL) *,†	799 (258–1759)	2360 (514–5511)	0.008
Elevated NT-proBNP (%) *,†	34 (51.5)	32 (65.3)	0.18
Cardiac TnI (ng/L) *,‡	9 (3.0–34.0)	8 (3.0–78.3)	0.22
Elevated cardiac TnI (%) *,‡	9 (20.9)	16 (33.3)	0.24
Heart rate (bpm)	82.4 ± 18	86.8 ± 21	0.010
Systolic blood pressure (mmHg)	127.1 ± 21	126.1 ± 24	0.59
Diastolic blood pressure (mmHg)	76.3 ± 13	74.5 ± 14	0.11
SpO_2_ (%)	91.9 ± 7	94.2 ± 4	<0.001

NT-proBNP = N-Terminal Pro-B-Type Natriuretic Peptide; SpO_2_ = peripheral capillary oxygen saturation. TnI = troponin I; * = Median (interquartile range); † = Data available for 66 COVID-19 and 49 no-COVID-19 patients only; ‡ = Data available for 43 COVID-19 and 48 no-COVID-19 patients only.

**Table 2:** Main ECG findings of COVID-19 and no-COVID-19 patients.

	COVID-19 Group (*n* = 324)	No-COVID-19 Group (*n* = 232)	*p*-Value	*p*-Value *
*Rhythm*			0.021	0.91
Sinus rhythm (%)	304 (93.8)	204 (87.9)		
Atrial fibrillation/flutter (%)	20 (6.2)	28 (12.1)		
PR interval (ms)	160.0 ± 31	163.8 ± 39	0.24	-
QRS duration (ms)	99.1 ± 18	99.9 ± 19	0.62	-
cQT interval	407.2 ± 29	401.8 ± 28	0.030	0.23
Prolonged cQT interval (%)	18 (5.6)	9 (3.9)	0.43	-
*Atrio-ventricular conduction*				
First-degree AV block (%)	21 (6.9)	22 (10.8)	0.14	-
*Intra-ventricular conduction*				
LBBB (%)	6 (1.9)	14 (6.0)	0.011	1.00
RBBB (%)	25 (7.7)	26 (11.2)	0.18	-
QRS ≥ 110 ms (%)	58 (17.9)	51 (22.0)	0.24	-
PSVCs (%)	11 (3.6)	17 (8.3)	0.029	0.59
PVCs (%)	13 (4.0)	13 (5.6)	0.42	-
*ST-segment/T wave abnormalities*				
STD ≥ 0.5 mm (%)	17 (5.3)	9 (4.1)	0.68	-
STD ≥ 1 mm (%)	4 (1.3)	7 (3.2)	0.13	-
T-wave inversion (%)	13 (4.1)	9 (4.1)	1.00	-
STD_0.5_/T changes (%)	28 (8.8)	16 (7.3)	0.63	-
STD_1.0_/T changes (%)	17 (5.3)	14 (6.4)	0.71	-
Abnormal Q/QS waves (%)	14 (4.4)	14 (6.4)	0.33	-
Any ECG abnormality (%)	120 (37.0)	101 (43.5)	0.13	-

AV = atrioventricular; ECG = electrocardiogram; LBBB = left bundle branch block; RBBB = right bundle branch block; cQT = corrected QT interval; PSVCs = premature supraventricular complexes; PVCs = premature ventricular complexes; STD = ST-segment depression. *p*-value * = *p*-values adjusted for age, sex, known heart disease, co-morbidity, hemoglobin, heart rate, and SpO_2_.

**Table 3:** Main clinical and ECG findings of dead and alive patients in the whole population of acute infectious respiratory disease (AIRD) patients.

	Dead (*n* = 81)	Alive (*n* = 475)	HR	95% C.I.	*p*-Value
COVID-19 diagnosis (%)	51 (63.0)	273 (57.5)	1.22	0.77–1.91	0.39
*Clinical findings*					
Age, years	79.2 ± 9	65.1 ± 16	1.07	1.05–1.09	<0.001
Male sex (%)	57 (70.4)	284 (59.8)	1.53	0.95–2.46	0.082
Known heart disease (%)	37 (45.7)	98 (20.6)	2.84	1.83–4.40	<0.001
Co-morbidity (%)	57 (70.4)	202 (42.5)	3.00	1.86–4.84	<0.001
Hypertension (%)	62 (76.5)	229 (48.2)	3.23	1.93–5.41	<0.001
Diabetes mellitus (%)	13 (16.0)	57 (12.0)	1.38	0.76–2.50	0.28
Dyslipidemia (%)	26 (32.1)	86 (18.1)	2.04	1.28–3.25	0.003
Smoking (%)	23 (28.4)	123 (25.9)	1.10	0.68–1.78	0.71
Hemoglobin, g/dL	12.6 ± 2.3	13.2 ± 2.2	0.90	0.82–0.99	0.039
Creatinine, mg/dL	1.79 ± 1.5	1.14 ± 1.3	1.18	1.09–1.27	<0.001
C-reactive protein, mg/L *	153 (90–193)	62 (19–146)	1.01	1.00–1.01	<0.001
Heart rate, bpm	91.1 ± 26	83.1 ± 18	1.02	1.01–1.03	<0.001
Systolic BP, mmHg	119.8 ± 25	127.9 ± 22	0.98	0.97–0.99	0.003
Diastolic BP, mmHg	70.2 ± 14	76.4 ± 13	0.97	0.95–0.98	<0.001
SpO_2_, %	87.7 ± 10	93.8 ± 5	0.93	0.91–0.94	<0.001
NT-proBNP, pg/mL *,†	2012 (1055–7814)	613 (195–2380)	1.00	1.00–1.00	<0.001
Elevated NT-proBNP (%) *,†	29 (85.3)	37 (45.7)	5.30	2.05–13.7	0.001
Cardiac TnI, ng/ mL*,‡	66.0 (17.0–228.0)	7.0 (3.0–34.8)	1.00	1.00–1.00	0.41
Elevated cardiac TnI (%) *,‡	8 (61.5)	17 (21.8)	4.87	1.59–14.9	0.006
*ECG findings*					
Atrial fibrillation (%)	13 (16.0)	35 (7.4)	2.25	1.24–4.07	0.007
PR interval, ms	173.0 ± 36	159.8 ± 34	1.01	1.01–1.01	0.003
LBBB (%)	5 (6.2)	15 (3.2)	1.99	0.81–4.93	0.13
RBBB (%)	12 (14.8)	39 (8.2)	1.88	1.02–3.47	0.044
QRS, ms	107.2 ± 24	98.1 ± 17	1.02	1.01–1.03	<0.001
QRS ≥ 110 ms (%)	26 (32.1)	83 (17.5)	2.13	1.33–3.39	0.002
cQT interval, ms	404.5 ± 36	405.0 ± 28	1.00	0.99–1.01	0.83
Abnormal cQT (%)	7 (8.6)	20 (4.2)	1.91	0.88–4.14	0.10
PSVCs (%)	7 (10.3)	21 (4.8)	2.28	1.04–4.99	0.039
PVCs (%)	6 (7.4)	20 (4.2)	1.75	0.76–4.01	0.19
STD ≥ 0.5 mm (%)	14 (18.4)	12 (2.6)	5.85	3.27–10.4	<0.001
STD ≥ 1 mm (%)	6 (7.9)	5 (1.1)	5.26	2.28–12.1	<0.01
T-wave inversion (%)	5 (6.6)	17 (3.7)	1.78	0.72–4.41	0.21
STD_0.5_/T changes (%) (%)	17 (22.4)	27 (5.9)	3.89	2.27–6.68	<0.001
STD_1.0_/T changes (%) (%)	10 (13.2)	21 (4.6)	2.78	1.43–5.41	0.003
Abnormal Q/QS wave (%)	9 (11.8)	19 (4.1)	2.95	1.47–5.92	0.002
Any ECG abnormality (%)	58 (71.6)	163 (34.3)	4.34	2.67–7.03	<0.001

BP = blood pressure; cQT = corrected QT; ECG = electrocardiogram; LBBB = left bundle branch block; NT-proBNP = N-Terminal pro-B-type natriuretic peptide; PSVCs = premature supraventricular complexes; PVCs = premature ventricular complexes; RBBB = right bundle branch block; SpO_2_ = peripheral oxygen saturation; STD = ST-segment depression; TnI = troponin I. * = Median (interquartile range); † = Data available for 34 dead and 81 alive patients, respectively; ‡ = Data available for 13 dead and 78 alive patients, respectively.

**Table 4:** Main results of multivariable survival Cox regression analysis in the whole population.

	Hazard Ratio	95% Confidence Interval	*p*-Value
Age	1.07	1.05–1.09	<0.001
C-reactive protein	1.01	1.00–1.01	<0.001
Creatinine	1.22	1.08–1.38	0.001
SpO_2_	0.94	0.91–0.96	<0.001
Co-morbidity	1.82	1.08–3.06	0.024
QRS duration	1.01	1.00–1.02	0.016
STD ≥ 0.5 mm	2.26	1.17–4.40	0.016
Any ECG abnormality	2.34	1.36–4.02	0.002

ECG = electrocardiogram; SpO_2_ = peripheral capillary oxygen saturation; STD = ST-segment depression.

**Table 5:** Main results of survival multivariable survival Cox regression analysis in the 2 groups of patients.

	COVID-19			No-COVID-19		
	Hazard Ratio	95% Confidence Interval	*p*-Value	Hazard Ratio	95% Confidence Interval	*p*-Value
Age	1.08	1.05–1.11	<0.001	1.09	1.04–1.13	<0.001
C-reactive protein	1.01	1.00–1.01	0.005	1.01	1.00–1.01	0.002
Creatinine	1.27	1.11–1.45	<0.001	-	-	-
SpO_2_	0.94	0.91–0.96	<0.001	-	-	-
Co-morbidity	2.08	1.14–3.80	0.017	-	-	-
LBBB	3.78	1.20–11.9	0.023	-	-	-
QRS duration	1.02	1.01–1.03	0.003	-	-	-
QRS ≥ 110 ms	1.85	1.02–3.35	0.044	-	-	-
STD ≥ 0.5 mm	1.79	0.81–3.93	0.15	4.64	1.61–13.3	0.004
STD ≥ 1.0 mm	2.85	0.65–12.4	0.16	4.51	1.44–14.1	0.010
STD_0.5_/T changes	2.16	1.06–4.42	0.034	1.64	0.59–4.56	0.34
STD_1.0_/T changes	2.20	0.89–5.43	0.086	1.38	0.46–4.14	0.57
Any ECG abnormality	2.16	1.12–4.20	0.022	2.83	1.06–7.53	0.037

ECG = electrocardiogram; LBBB = left bundle branch block; SpO_2_ = peripheral capillary oxygen saturation; STD = ST-segment depression.
